# The significance of mosquito saliva in arbovirus transmission and pathogenesis in the vertebrate host

**DOI:** 10.1016/j.onehlt.2023.100506

**Published:** 2023-02-12

**Authors:** Imke Visser, Constantianus J.M. Koenraadt, Marion P.G. Koopmans, Barry Rockx

**Affiliations:** a Department of Viroscience, Erasmus University Medical Center, Rotterdam, the Netherlands; b Laboratory of Entomology, Wageningen University & Research, Wageningen, the Netherlands

**Keywords:** Arbovirus, Mosquito saliva, Transmission, Pathogenesis

## Abstract

Due to changes in climate, numerous mosquito species are continuously extending their geographical distributions, posing potential new public health threats as arbovirus infections emerge in these new areas. During probing and feeding on the vertebrate host, a mosquito can inject both arbovirus and saliva into the skin of the host. The presence of mosquito saliva in the host skin during arbovirus transmission contributes to high viral titers in the skin, enhanced viremia, and rapid dissemination of the virus to target organs. This enhanced phenotype effectuated by the presence of mosquito saliva in the skin can be partly ascribed to a polarization of the local immune balance towards a Th2 response, an increased permeability of the dermal endothelium, and the influx of virus-susceptible immune cells to the bite site. However, the complete identification and characterization of immunomodulatory salivary proteins from different mosquito species and the mechanisms by which these salivary proteins exert their effects synergistically or antagonistically remains to be further explored. Moreover, the effect of new virus-vector combinations on the outcome of arbovirus infection in a new host is limited. Here, we review the immunomodulatory effects of mosquito saliva in the skin and the proposed mechanisms by which mosquito saliva enhances arbovirus pathogenesis in the vertebrate host, and discuss potential differences between *Aedes* and *Culex* mosquito species, the main vectors for medically important arboviruses. Gaining more insight into the effect of mosquito saliva in the vector-virus-host triad aids in predicting the potential transmission risk and disease severity of emerging vector-borne diseases.

## 1 Arboviruses and their mosquito vectors

Arthropod-borne (arbo) viruses comprise a range of different virus families and are transmitted primarily by arthropod vectors such as mosquitoes or ticks. Approximately 3.9 billion people in the tropics and sub-tropics are at risk of arboviral infections. Medically important mosquito-borne viruses include yellow fever virus (YFV), Zika virus (ZIKV), dengue virus (DENV), chikungunya virus (CHIKV), and West Nile virus (WNV) [1]. Mosquito species that play a major role in the transmission of these arboviruses include *Aedes* (*Ae.*) *aegypti* and *Ae. Albopictus* as well as *Culex* (*Cx.*) species. Arbovirus infection in humans is often asymptomatic but can lead to serious disease including encephalitis, arthralgia, haemorrhagic fever, and death [2].

Over the past few decades, numerous mosquito species have expanded their geographical range due to climate change, deforestation, urbanisation, increased travel and global trade [[3], [4], [5], [6], [7], [8], [9], [10]]. For example, the origins of *Ae. aegypti* and *Ae. albopictus* trace back to tropical forest areas. However, these species are now established throughout the world, in particular Brazil and the USA, but there are also occurrences in Asia, Africa, Oceania, and southern Europe [7,[11], [12], [13], [14]]. Regular incursions of these mosquito species are reported in non-endemic areas, including the Netherlands. While it is unlikely for *Ae. aegypti* to become established in northern latitudes with temperate climates in the near future, such as north-western Europe [9,15], *Ae. albopictus* is more tolerant to colder temperatures and is capable of readily adapting to new (man-made) environments [6,14,[16], [17], [18], [19], [20]], thus could potentially establish itself in northern latitudes [6,16,17].

Simultaneously, arboviruses are emerging in areas where suitable mosquito vectors are already present, such as the recent emergence of WNV in north-western Europe, vectored by *Cx. pipiens* (also known as the common house mosquito) [21]. WNV is now widespread in Europe and causes neuroinvasive disease in humans. An arbovirus related to WNV is the less-studied Usutu virus (USUV), which shares a similar transmission cycle between vectors and birds as their reservoir host species as WNV. USUV first emerged in 2001 in Austria [22], but has since caused mass die-offs in birds in the majority of Western European countries, including recent outbreaks in the Netherlands, Belgium, France and Germany in 2016–2018, and continues to spread across Europe [23].

The main vector of both WNV and USUV is *Cx*. *pipiens*. *Culex spp.* mosquitoes are distributed throughout the world and are primary vectors for a wide array of (neurotropic) arboviruses including WNV, USUV, and Japanese encephalitis virus (JEV). However, other mosquito species are also suggested to be competent vectors for WNV, including *Ae. albopictus* [24,25]. The opportunistic feeding behaviour of *Ae. albopictus* biting both mammals and birds may render this mosquito species an excellent bridge vector transferring endemic arboviruses such as WNV from a sylvatic cycle to the human population [26,27]. In addition, the possibility of arboviruses to naturally adapt to a new mosquito species and expand their global distribution cannot be ruled out. For example, a single mutation in the CHIKV genome shifted its specificity from its typical vector *Ae. aegypti* to *Ae. albopictus*, leading to CHIKV outbreaks in areas where *Ae. aegypti* is absent [28,29].

During arbovirus transmission, the mosquito bite itself is of crucial importance; the saliva injected in the skin during the bite can markedly shape the establishment of arbovirus infection and disease development in the vertebrate host [30]. For example, the presence of mosquito saliva during arbovirus infection enhances or prolongs viremia in *in vivo* studies when compared to inoculation of virus alone [[31], [32], [33], [34], [35], [36], [37], [38], [39]]. An alteration in host viremia could have implications for the transmission dynamics of circulating arboviruses. Higher host viremia levels increases the chances of a mosquito to pick up the virus while taking a bloodmeal and subsequently transmit the virus to a new host [40,41].

While mosquito saliva is naturally present during arbovirus transmission from the mosquito vector to humans, it is unknown whether saliva from exotic mosquito species that are not primarily associated with specific endemic arboviruses, differentially affect host viremia and clinical outcome. This is of concern, considering the potential for new combinations of vectors and viruses due to their geographic expansion. The virus-enhancing effect of mosquito saliva has been most extensively studied for *Ae. aegypti*, possibly due to the fact that it is the primary vector for arboviruses that are affecting the highest number of people worldwide [42]. Regardless, the effect of mosquito saliva from *Ae. albopictus* has only once been included in a recent study, despite the fact that it can also be considered a primary vector for medically important arboviruses such as DENV and CHIKV [43]. In addition, there are only a few studies where the effect of saliva from different *Aedes* and *Culex* mosquito species were compared side by side [34,35]. It is therefore unknown whether the effects on pathogenesis of arboviruses are a general feature of saliva for all haematophagous mosquito species.

Here we reviewed the current knowledge on the effects of mosquito saliva on arbovirus transmission and pathogenesis and identified key gaps in knowledge. For this review paper, the PubMed and Scopus databases were used and we included the following search terms: [Vector OR Culex OR Aedes OR Anopheles]; [Arbovirus OR flavivirus OR mosquito-borne virus OR arthropod-borne virus]; [Transmission OR mosquito bite]; [Skin OR dermis OR skin cells]; [Immune system OR immune cells OR immunity]; [Mosquito saliva OR salivary proteins OR mosquito bite]; [Europe]; [Climate change] AND [Vector competence] AND [mosquito feeding behaviour] AND [temperature];[Pathogenesis OR tissue tropism OR neuroinvasion];[Mosquito bite AND allergy]. Exclusion criteria included non-English written papers; papers not focussing on arboviruses.

## 2 Establishment of infection and antiviral responses in the skin

During arbovirus transmission, mosquitoes deposit virus-loaded saliva into the skin while probing and feeding [44]. The skin serves as the initial site of arbovirus replication prior to the virus reaching the bloodstream and disseminating to other organs. The skin consists of the dermis and epidermis and is composed of different non-hematopoietic skin cells and skin-resident immune cells. The main cellular components of the skin are keratinocytes in the granular layer of the epidermis [45,46] and fibroblasts in the dermal layer. Mosquitoes are able to probe through the entire dermis up until the hypodermis (the fat layer), where bites are detectable as small haemorrhagic spots [47].

Arboviruses are able to infect a range of skin cells including keratinocytes [[48], [49], [50], [51], [52], [53]], fibroblasts [48,50,54,55], (immature) dendritic cells (DCs) [48,52,56,57], Langerhans cells (DC population which resides in the epidermis) [52,[57], [58], [59], [60]], mast cells [52,61], and macrophages [52,56,57,59]. Cells involved in the skin immune system include macrophages, neutrophils, DCs, mast cells, and lymphocytes [46,62,63], and aid in protecting the host from microbial pathogens and allergens. However, non-immune skin cells like keratinocytes also exert immune-regulating effects upon infection [52,64] by expressing pathogen recognition receptors such as toll-like receptor 3/7 [[65], [66], [67]] and interacting with skin-resident immune cells to induce immune responses [68]. Upon recognition of viral RNA by endosomal RNA sensors, virus infection generally triggers host innate immune responses to rapidly control viral replication and spread [[69], [70], [71], [72], [73], [74]]. For example, the expression of interferon (IFN)ß [50,54,75] and tumor-necrosis factor (TNF)α [54] is upregulated in fibroblasts and keratinocytes upon infection [50,66]. In fact, keratinocytes are thought to play a valuable role in inciting cutaneous inflammation [45,76]. Infection of keratinocytes leads to an increased production of cytokines interleukin (IL)1ß [52,77], IL6, TNFα [78], IFNß, IFNγ [49], and chemokines CXCL-1, 2, 8, 10, and CCL20 [78] which are critical for recruiting local immune cells and establishing an antiviral immune state shortly after an infectious mosquito bite.

Activated Langerhans cells are able to extend their dendrites up until right below the stratum corneum (the outermost layer of the epidermis), penetrating keratinocyte tight-junctions to scan for, and take up, external antigens [79] followed by maturation into potent immunostimulatory DCs [53,80,81]. Langerhans cells require signals from IL1ß [82] and TNFα [83] for migration to the draining lymph nodes (dLNs) [52,59,84,85] to present viral antigen [86,87], followed by a leukocyte influx into the dLNs [87,88]. Langerhans cell-susceptibility to arbovirus infection coincidentally allows virus migration to the dLNs [60] and consequent viral spread to distant organs. Likewise, infection of dermal DCs and macrophages leads to the recruitment of monocytes from the blood to the dermis, which subsequently differentiate into DCs that can also become infected and migrate to the dLN [56,57].

Another route arboviruses may take to travel to the dLNs is *via* infection of mast cells and subsequent transport from the infection site to the dLNs in extracellular mast cell granules, although this has so far only been studied for DENV [61]. In addition, infected mast cells signal to dermal endothelial cells to increase the expression of intercellular adhesion molecule and vascular cell adhesion molecule [61], which mediate the adhesion and migration of leukocytes through the endothelium of blood vessels [89]. Neutrophils are one of the first immune cells recruited to the site of infection [90] but may be susceptible to infection, as is shown for WNV [91]. The recruitment of immune cells to the bite site may thus inadvertently provide extra targets for arbovirus replication in the skin and migration to the dLNs and beyond.

Considering that dermal cells initiate antiviral immune responses but simultaneously facilitate viral replication and systemic spread, the initiation of an inflammatory response can result in both a protective or pathogenic outcome [92]. Efficient early peripheral replication contributes to the capacity of neurotropic arboviruses to cause neuroinvasion and mortality [69,93,94]. As such, the dampening of antiviral T-helper (Th)1 responses in the skin following a mosquito bite creates an immune environment that partly favours peripheral viral replication before dissemination to major target organs such as, in case of neurotropic arboviruses, the brain.

## 3 Mosquito saliva: Skewing the immune balance

Mosquitoes probe their host for 1–7 min depending on the mosquito species [44,95], mosquito age and infection status [47], and host species [95]. Mosquito saliva is retained in the host's skin for 4–18 h after feeding [47,96], where it initially exerts vasodilatory and anti-coagulatory functions to aid the mosquito in successfully taking up a blood meal straight from a capillary or from resulting blood pools [30,47,95,[97], [98], [99], [100]]. The skin is rich in capillaries, veins, and arteries and when stimulated by mosquito saliva dermal microvascular endothelial cell permeability is induced. This results in plasma extravasation [101,102] and the ensuing appearance of oedema following the bite of a mosquito [103]. This is regulated by mast cell activation and degranulation [101], the subsequent release of histamine [102], or through a direct effect of mosquito saliva [104,105]. Concurrently, mosquito saliva polarizes the skin towards a Th2 immune response as it induces the production of high levels of IL4 [33,[106], [107], [108]] and IL10 [33,107,109,110], along with a decreased amount of IFNß [109], and IFNγ [107,108,110,111]. A Th2-dominated immune milieu at the bite site results in a classic type I allergic reaction mediated by IgE [112,113], IL10, and mast cells [110,114].

The presence of mosquito saliva at the bite site promotes homing of immune cells to the skin and includes eosinophils, monocytes, mast cells, CD4+ T-cells [115], and neutrophils [101,103,109,115]. Recruited neutrophils initiate innate immune responses and express the chemoattractant CXCL2, which stimulates the migration of monocytic cells from the bloodstream into the skin [103]. Under the influence of local inflammatory cytokines, monocytes differentiate into macrophages and DCs [116]. Mosquito saliva consists of a myriad of different proteins for many of which the immunomodulatory properties still need to be elucidated. Only a subset of specific salivary proteins, mostly those of *Ae. aegypti*, have been studied *in vivo* for their effect on arbovirus pathogenesis [[117], [118], [119], [120], [121], [122], [123]] (Table 1). For example, the *Ae. aegypti* salivary protein NeSt1 induces IL1ß and CXCL2 expression at the inoculation site, which activates neutrophils, induces macrophage infiltration into the bite site, and enhances viral pathogenesis [117]. Likewise, the *Ae. aegypti* salivary protein SAAG-4 reduces *in vitro* CD4+ T-cell expression of IFNγ while simultaneously programming T-cells to express the Th2 cytokine IL4 [124], which creates a Th2-dominant environment that can further stimulate naïve CD4+ T-cells to differentiate into Th2 cells [125].

**Table 1:** Specific salivary proteins expressed in the salivary glands of *Ae. aegypti* that are studied for their effect on arbovirus pathogenesis *in vivo* (mice). ZIKV = Zika virus, DENV = dengue virus, SFV = Semliki forest virus.

Salivary factor	Effect *in vivo*	Proposed mechanism	Reference
*Aedes aegypti* Venom allergen-1 (*Aa*VA-1)	Promotes ZIKV and DENV infection	Activation of immune cell autophagy	[121]
LTRIN	Enhanced ZIKV pathogenesis	Inhibiting LTβR signalling	[118]
Neutrophil stimulation factor 1 (NeSt1)	Enhanced ZIKV pathogenesis	Activation of neutrophils and recruitment of macrophages to the bite site	[117]
*Ae. aegypti* bacteria-responsive protein 1(AgBR1)	Enhanced ZIKV pathogenesis	Induction of neutrophil infiltration to the bite site	[122]
Aegyptin	Lower DENV pathogenesis	Augmentation of cytokine concentrations in the inoculation site	[119]
Sialokinin	Enhanced SFV pathogenesis	Induction of blood vascular barrier leakage	[105]

Of note, mosquito salivary protein transcripts are differentially expressed upon blood meal digestion, as opposed to sugar feeding. Some salivary gland proteins are constitutively expressed, but blood-feeding *versus* sugar-feeding modulates the expression levels [126,127]. Their activity can be either abrogated [128] or induced upon blood feeding [118,129], suggesting that the feeding status of a mosquito can influence the immunomodulatory properties of mosquito saliva as a whole. For example, *Aedes* D7 proteins and apyrase are upregulated upon blood-feeding [126]. The D7 proteins of *Ae. albopictus* and *Cx. quinquefasciatus* inhibit the recruitment of eosinophils and neutrophils [130], and facilitate blood feeding to the mosquito by inhibiting platelet aggregation [100,130] and antagonizing vasoconstriction [131]. Apyrase is an enzyme that inhibits platelet aggregation during blood-feeding [132] and prevents neutrophil activation [133]. Also the activity of *Ae. aegypti* salivary enzyme adenosine deaminase is upregulated in the salivary glands after a blood meal. Upregulation of its activity could lead to the inhibition of platelet aggregation, inhibition of proinflammatory cytokine production, and inhibition of mast cell degranulation [126,134]. Adenosine deaminase is known to be present in the salivary glands of *Ae. aegypti* and *Cx. quinquefasciatus*, and it appears that only *Ae. aegypti* secretes adenosine deaminase in its saliva [134]. Almost all proteins that are upregulated in the salivary glands of blood-fed mosquitoes seem to have an important role in successful blood-feeding. The proteins that are downregulated in blood-fed mosquitoes (and upregulated in sugar-fed mosquitoes) tend to have housekeeping functions [126]. On the whole, the immunogenic properties of many mosquito salivary proteins remain undetermined, including any possible synergistic or antagonistic effects salivary proteins might exert at the vector-host interface.

## 4 Effect of mosquito saliva on arbovirus pathogenesis

A substantial amount of *in vivo* data, using experimental mouse models, shows that co-inoculation of virus with mosquito saliva, inoculation *via* an infectious mosquito bite, or feeding of uninfected mosquitoes prior to virus inoculation generally leads to a higher virus titer in the skin [31,38,103,104,129], higher and/or longer-lasting viremia [[31], [32], [33], [34], [35], [36], [37], [38], [39]], higher tissue titers and/or earlier spread to other tissues [31,38,39,103], and higher or accelerated mortality rates [31,35,38,103,104,121,135] compared to needle-inoculation. The bite of even one mosquito already enhances viral infection when compared to needle-inoculation [37,39], however, enhanced viremia is sustained for a longer time when mice are probed by more mosquitoes [37]. The effect of mosquito saliva is dose-dependent [37] and local, meaning that mosquito saliva deposited away from the bite site does not augment viral pathogenesis [34,37,39]. It is also timing-dependent; mosquito saliva enhances viremia when injected from 24 h before to 12 h after virus inoculation [37].

Most virus is injected extravascularly during probing and feeding by the mosquito [44,[136], [137], [138]]. This initially leaves the virus confined to the bite site rather than rapidly disseminating *via* the circulatory system [136] following the bite of a mosquito [103,139]. Surgical removal of the virus inoculation site in the absence of mosquito saliva improves survival chances of the host [136], an effect that is achieved up until (at least) 4 h after virus inoculation [104]. However, when *Ae. aegypti* saliva is present at the bite site, removal of the skin 4 h after inoculation does not have any protective effect, suggesting that arboviruses disseminate to the dLNs and beyond more rapidly in the presence of mosquito saliva at the bite site [104]. In contrast, another study found that the presence of *Ae. aegypti* saliva during arbovirus infection results in a higher viral load in the skin in conjunction with significantly lower virus titers in the dLNs 3 and 6 h post-infection, yet from 24 h onwards the opposite is observed. Furthermore, the presence of saliva results in earlier and higher viral titers in remote LNs, *i.e.* away from the bite site, as well as in the brain, compared to inoculation of virus alone [103]. This indicates that the enhancing effect of mosquito saliva is not attributed to early rapid dissemination of virus from the skin to the dLNs but rather suggests retention and efficient replication of the virus at the bite site, before subsequently disseminating to remote LNs and organs.

**Fig. 1:**
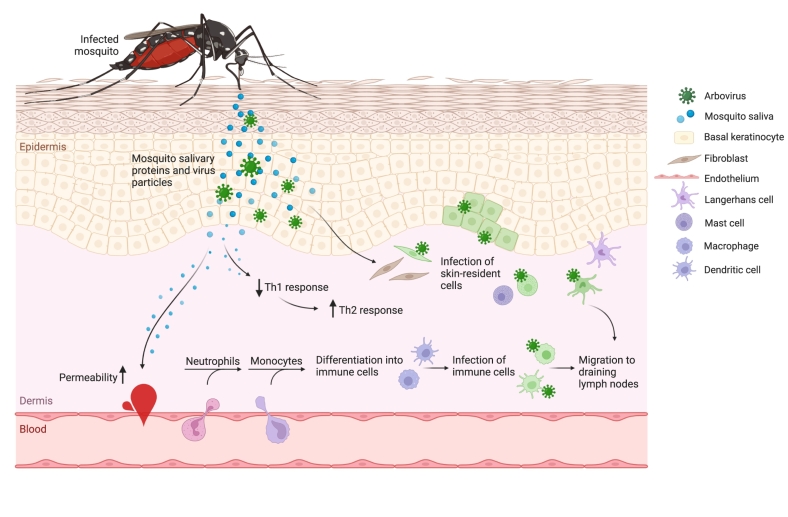
Arbovirus transmission from the mosquito vector to a vertebrate host. Schematic overview of the early events in the skin upon the bite of an infectious mosquito. During probing and feeding, a mosquito injects both saliva and virus particles into the host skin. Arboviruses infect a range of resident skin-cells including dermal fibroblasts, epidermal keratinocytes, mast cells, and Langerhans cells. Simultaneously, mosquito salivary proteins induce permeability of the endothelium of dermal capillaries while also dampening antiviral Th1 immune responses, resulting in a local Th2-dominant immune response. Both virus infection in the skin and an increased permeability of the endothelium allows for an influx of neutrophils to the bite site followed by an influx of monocytes, where these cells differentiate into dendritic cells or macrophages. Subsequently, the infected immune cells in the skin migrate to the draining lymph nodes followed by dissemination to distant organs. Green cells represent infected cells.

Neutrophil recruitment to the bite site, brought about by the presence of *Ae. aegypti* saliva, is observed as early as 3 h post-bite along with an increased level of dermal vascular leakage [103], while an influx of monocytic cells is seen between 2 and 16 h post-bite [104,105]. These findings suggest that the mechanism by which viruses disseminate to distant organs more rapidly due to the presence of mosquito saliva at the bite site partially occurs through first confining the virus at the bite site for (at least) 6 h. This is followed by increased viral titers in the skin as a result of the influx of neutrophils and (susceptible) myeloid cells 3 to 16 h post-bite. Subsequently, the virus disseminates to the dLNs, remote LNs, and distant organs [103]. This likely occurs in combination with hampering early viral clearance through the downregulation of Th1 cytokines, shifting the immune balance towards a Th2 response [107,140], and aided by an increase in dermal microvascular permeability [101,104,105] (Fig. 1). Overall, an alteration in immune cell populations as well as cytokine and chemokine signalling effectuated by the presence of mosquito saliva in the skin contributes to the dysregulation of antiviral signalling by antigen-presenting cells, ultimately influencing arbovirus pathogenesis [109].

Collectively, the most studied vector-virus pairing *in vivo* is DENV in combination with *Ae. aegypti* (Table 2), where it is repeatedly shown that DENV pathogenesis is enhanced when transmitted *via* infectious *Ae. aegypti* bites [32,33,141], pre-exposure to *Ae. aegypti* probing prior to virus inoculation [36], or co-inoculation of *Ae. aegypti* saliva and virus [104]. The effect of *Ae. albopictus* or *Ae. japonicus* saliva on DENV pathogenesis *in vivo* remains unexplored, even though both species are considered competent vectors for DENV [43,142]. It is possible that the *Ae. aegypti* salivary proteins responsible for the observed enhanced DENV pathogenesis are conserved within the *Aedes* genus or even across species [131,143], which would allow extrapolation of data from studies with *Ae. aegypti* and DENV to other arbovirus pathogenesis-enhancing effects that saliva of other members from the *Aedes* (or even *Culex*) genus might have. This is supported by the comparable effects of saliva from *Cx. tarsalis* [37,39,129] and *Ae. aegypti* [38,109,135] on WNV infection in mice, where the presence of saliva results in enhanced viremia, higher viral load at the inoculation site, and earlier neuroinvasion. Likewise, Cache valley virus viremia is enhanced to the same extent by saliva from *Ae. aegypti*, *Ae. triseratius*, or *Cx. pipiens* [34]. However, while saliva of both *Ae. triseriatus* and *Ae. aegypti* increase Rift valley fever virus (RVFV) tissue titers, viremia, and mortality rates in a uniform manner, saliva of *Cx. pipiens* does not have an enhancing effect on RVFV infection [35]. Therefore, differences in the effect of mosquito saliva on arbovirus pathogenesis may indeed differ among species. Whether the effects of mosquito saliva on the pathogenesis of distinct (arbo)viruses differs also remains a gap in knowledge, for example there is so far no data available on the effect of mosquito saliva on the pathogenesis of JEV and USUV in a vertebrate host.

**Table 2:** Overview of available *in vivo* data on different vector-virus pairings studying the effect of mosquito saliva on arbovirus pathogenesis. Includes data from either an infectious mosquito bite, saliva co-inoculation with virus, or feeding/probing of uninfected mosquitoes prior to virus inoculation. Cx. = Culex, Ae. = Aedes. WNV = West Nile virus, ZIKV = Zika virus, DENV = dengue virus, JEV = Japanese encephalitis virus, USUV = Usutu virus, CHIKV = chikungunya virus, SFV = Semliki forest virus, VEEV = Venezuelan equine encephalitis virus, RVFV = Rift valley fever virus, LACV = La Crosse virus, CVV = Cache Valley virus. (+) indicates enhanced pathogenesis, referring to one or more of the following parameters: higher (early) and/or longer-lasting viremia, higher viral load at inoculation site and/or remote tissues, earlier neuroinvasion, higher or accelerated mortality rates, increased morbidity. (-) indicates no effect on pathogenesis, blank indicates no *in vivo* data available on this vector-virus pairing.

		***Ae. aegypti***	***Ae. albopictus***	***Ae. vexans***	***Ae. triseriatus***	***Ae. taeniorhynchus***	***Cx. pipiens***	***Cx. tarsalis***	***Cx. quinquefasciatus***	References
*Flaviviridae* Genus ***Flavivirus***	**WNV**	**+**						**+**	**-**	[37,38,39,130,135,147]
	**ZIKV**	**+**	**+**				**+**			[105,117,118,121]

**DENV**	**+**								[32,33,36,104,129,141]

*Togaviridae* Genus ***Alphavirus***	**CHIKV**	**+**								[31]
	**SFV**	**+**	**+**				**+**			[103,105]

**VEEV**					**-**				[136]

*Bunyaviridae* Genus ***Phlebovirus***	**RVFV**	**+**		**+**			**-**			[35]

*Bunyaviridae* Genus ***Orthobunyavirus***	**LACV**				**+**					[148]
	**CVV**	**+**			**+**		**+**			[34]

In addition to the mosquito salivary proteins that enhance arbovirus pathogenesis, some salivary proteins may in fact protect the host against development of arbovirus disease. The presence of mosquito salivary protein D7 can inhibit DENV infection [144], and neutralizing the D7 protein through vaccination resulted in enhanced mortality after WNV infection in mice [120]. The D7 salivary protein family is conserved across mosquito species (*Aedes*, *Culex*, and *Anopheles*) and other blood-feeding insects such as sandflies [131]. In addition to modulating the host response, factors within mosquito saliva have also been shown to interact with the virus directly, affecting its infectivity. The D7 protein of *Ae. aegypti* inhibits DENV infection in mice, possibly through the direct interaction of D7 with the envelope protein of DENV [144]. In addition to D7 binding the DENV envelope protein, three other *Ae. aegypti* salivary proteins are shown to bind to the ZIKV envelope protein, two of which have anti-thrombotic or anti-platelet aggregation functions [145]. Although the exact mechanism by which the binding of mosquito salivary proteins to the virus envelope protein mediates viral infectivity is unknown, it may have implications for binding of the virus to host cell receptors.

## 5 Discussion and future perspectives

The transmission of arboviruses from a mosquito vector to a vertebrate host invariably involves mosquito saliva. Mosquito saliva consists of a cocktail of bioactive compounds that aid the mosquito in successfully taking up a blood meal through halting blood clotting of dermal vessels, inducing vasodilation and promoting cutaneous oedema [30,95,[97], [98], [99], [100]]. It is suggested that the extent of the host immune response following a mosquito bite partially dictates the severity of arboviral disease in the vertebrate host [103], however the detailed mechanism by which mosquito saliva enhances viral replication and pathogenesis remains to be further unravelled.

The most important parameter driving arbovirus outbreaks is suggested to be the host-feeding preference of mosquitoes, which is, among other things, dependent on the (seasonal) abundance of reservoir host species [149]. Most mosquito species that display a strong inherent anthropophilic host-preference belong to *Aedes spp.*, the vectors that account for transmitting nearly all medically important arboviruses to humans. It is therefore speculated that host-preference has co-evolved with the evolution of arboviruses with their host [150]. The salivary protein transcripts may thus vary between mosquito species showing distinct host-feeding preferences. For example, the blood clotting mechanism of birds is different from that of humans in terms of coagulation time, which is longer for birds compared to mammals [[151], [152], [153], [154]]. It may therefore be redundant for strictly ornithophilic mosquito species, such as some of those belonging to the *Culex* genus, to have evolved salivary factors that rapidly antagonize coagulation in order to facilitate blood meal acquisition.

*Aedes* mosquitoes have a longer evolutionary linkage with mammals compared to *Culex* mosquitoes [95]. As such, *Cx. quinquefasciatus* takes significantly more time finding blood when fed on a human forearm in comparison to *Ae. aegypti*, while there are no differences between these mosquito species in probing and feeding time when fed on a bird [95], indicating that *Culex* may indeed not possess a specific anti-clotting salivary protein that optimizes blood-feeding on mammals to the same degree as *Aedes*. Recently, an *Ae. aegypti*-specific salivary protein responsible for inducing dermal endothelial permeability in mice has been identified and no homologue of this protein was found in *Ae. albopictus*, *Cx. tarsalis* or *Cx. quinquefasciatus*. This finding implies that the identified salivary protein is *aegypti*-specific, rather than being specific for anthropophilic mosquito species. However, since both *Cx. pipiens* and *Ae. albopictus* enhance arbovirus infection *in vivo* to a similar amount as *Ae. aegypti* [105], they most likely possess other factors responsible for the observed enhanced phenotype *in vivo* (Table 2). For example, while the anti-clotting activity of *Cx. quinquefasciatus* saliva is significantly lower compared to *Ae. aegypti*, the anti-platelet activity is found to be the same for both species, while the vasodilatory activity is higher for *Cx. quinquefasciatus* than for *Ae. aegypti* [95]. Thus, although the salivary composition of *Culex* may not be optimally adapted to facilitate feeding on a mammalian hosts, more research into *Culex* immunomodulatory salivary factors is needed in order to identify and characterize the specific *Culex* salivary proteins that favour virus replication in a mammalian host.

One important detail to consider is the diverse methods used to isolate mosquito saliva for *in vitro* and *in vivo* assays in order to study its pathogenesis-enhancing properties. Most research groups either isolate pure mosquito saliva by employing a forced salivation assay using sugar water or immersion oil, or dissect and homogenize whole mosquito salivary glands. Crude salivary gland extracts presumably contain cellular compounds that in a natural setting would not be injected into the host during probing and feeding, and may therefore be considered a disadvantage of this method. In addition, for both assays it should be taken into account that mosquito salivary protein transcripts are differentially expressed upon blood meal digestion, as opposed to sugar feeding [126,127]. However, a recent paper found comparable enhancing effects *in vivo* of saliva from blood-fed *versus* sugar-fed *Ae. aegypti* [105]. Furthermore, an infected mosquito shows increased probing and biting behaviour [155,156] or changed salivary gland physiology [157], which may eventually increase arbovirus transmission rates [97]. Using uninfected mosquito saliva or probing prior to virus inoculation in an *in vivo* model may therefore not recapitulate what happens in nature and yield differential results compared to infecting an animal model *via* an infectious mosquito bite. However, when using infectious mosquitoes it is difficult to know the exact viral dose that is injected after a bite, since it was recently shown that the forced salivation assay that is broadly applied to assess viral load in mosquito saliva may underestimate the actual arbovirus load transmitted to a new host [158]. Overall, such aspects should be considered when interpreting data on the pathogenesis-enhancing properties of mosquito saliva.

Studies on the effect of mosquito saliva on arbovirus pathogenesis in a vertebrate host mainly focus on combinations of an arbovirus in combination with its primary vector, for example DENV and *Ae. aegypti*. However, numerous mosquito species are continuously expanding their geographical range, which results in new combinations of vectors and viruses. Therefore, the relative contribution of saliva from different mosquito species with regards to arbovirus transmission dynamics and transmission risk should be further elucidated when taking into account different vector-virus pairings. This review highlights a major gap in knowledge on the effects of mosquito saliva from exotic mosquito species on the pathogenesis of endemic viruses and *vice versa*. Studying this facet of arbovirus transmission could aid in predicting whether different vector-virus pairings will trigger clinical arbovirus disease or change its clinical manifestations in humans. In addition, studying the effect of mosquito saliva on arbovirus transmission will extend the existing vector competence studies as a risk assessment for potential arbovirus transmission or alteration in transmission dynamics. Another major gap in knowledge is the effect environmental (climate) changes may have on the composition of mosquito saliva and thereby its effect on transmission and pathogenesis. While it is known that external factors such as temperature and food abundance can affect mosquito development and host gene expression profiles, data on changes in salivary glands and subsequent saliva composition are largely unavailable. Moving forward, identification and characterization of novel salivary proteins from distinct mosquito species will advance the development of intervention methods such as the establishment of a mosquito saliva-based vaccine [159].

## Funding

This work is part of the research program One Health PACT with project number 109986, which is partly financed by the Dutch Research Council (NWO).

## Author statement

Individual contributions of authors to this review paper:

Imke Visser: Conceptualization, Visualization, Investigation, Methodology, Writing – original draft, review & editing.

Barry Rockx: Conceptualization, Writing – original draft, review & editing, Funding acquisition.

Constantianus J.M. Koenraadt: Writing – review & editing.

Marion P.G. Koopmans: Writing – review & editing, Funding acquisition.

## Declaration of Competing Interest

The authors have declared that no competing interests exist.

## Data Availability

No data was used for the research described in the article.
